# Biomechanical Behavior of Different Framework and Superstructure Material Combinations in Two-Implant-Supported Four-Unit Prostheses: A Dynamic Finite Element Analysis

**DOI:** 10.3390/ma19112376

**Published:** 2026-06-03

**Authors:** Niloofar Hajghani, Burcu Günal-Abdulcelil

**Affiliations:** Department of Prosthodontics, Faculty of Dentistry, Near East University, Near East Boulevard, Mersin 10, Nicosia 99138, Turkey; burcu.gunal@neu.edu.tr

**Keywords:** dynamic finite element analysis, dental materials, dental prosthesis, dental framework materials, implant

## Abstract

The long-term success of implant-supported prostheses (ISPs) is strongly influenced by material selection, which affects stress distribution within the implant system and surrounding cortical bone. This study aimed to assess the biomechanical behavior of a four-unit ISP supported by two implants in the posterior region, using different framework and superstructure material combinations through dynamic finite element analysis (FEA). **Methods:** A three-dimensional (3D) edentulous mandibular model was created using Mimics software, with two implants placed in the first premolar and second molar regions. Four framework materials—titanium (Ti), glass fiber–reinforced composite (GFRC), 3Y-TZP zirconia, and polyether ether ketone (PEEK)—were combined with two superstructure materials, 5Y-TZP zirconia and resin-matrix ceramic (RMC), forming eight groups. Dynamic loading simulated chewing forces, and stress distribution was analyzed using the von Mises criterion. **Results:** The results demonstrated that 3Y-TZP zirconia frameworks generated the highest stress values across implants, abutments, and cortical bone. RMC crowns consistently produced lower stress than 5Y-TZP zirconia across all the groups. PEEK showed the highest displacement, followed by GFRC, zirconia, and Ti. **Conclusion:** Materials with higher Young’s modulus tended to exhibit greater stress transfer to the implant, implant components, and cortical bone. In contrast, polymer-based materials may show a tendency toward greater deformation and displacement compared with metallic and ceramic materials.

## 1. Introduction

Implant-supported rehabilitations have demonstrated high success rates in both short- and long-term studies [1,2,3]. Implant-supported prostheses (ISP) provide biological and biomechanical advantages, such as preserving adjacent and opposing teeth, maintaining supporting bone structures, and enhancing masticatory function [4,5]. Multiple factors influence the success of ISP, including appropriate implant selection, the choice of prosthetic materials, prosthetic design [6,7], number of supporting implants [8], occlusal forces and occlusion [9], type of abutment connection [10], and density of the supporting bone [11].

The prosthetic design of an ISP plays a critical role in stress distribution and therefore influences its long-term success [6,12]. Although the initial stability of an implant depends on the quality and quantity of trabecular and cortical bone, improper distribution of occlusal forces can lead to implant failure despite optimal initial stability and osseointegration [13]. Due to the importance of prosthesis design, a different design of screw-retrievable and cement-retained ISP with the combined benefits of both forms of retention has been introduced by several authors [14,15,16]. This design is commonly referred to as a Toronto prosthesis. In this technique, a screw-retained framework supports individually cemented crowns, and pink or gingiva-colored porcelain or laboratory composite is used to mimic the surrounding soft tissues [14,15]. 

Various restorative materials have been introduced for use as framework materials in ISP. The framework material may have significant effects on stress transfer to the ISP and peri-implant bone region [17]. The framework supports the superstructures, minimizes the number of implants, improves load distribution, and decreases the stresses generated on bone and mucosa [18].

The favorable biocompatibility, relatively low cost, low density, and desirable mechanical properties of titanium (Ti) [17], together with the advantageous electrochemical behavior of Ti alloys under inflammatory conditions [19], make Ti a highly suitable material for prosthetic frameworks. However, metal frameworks also exhibit certain limitations, including the potential for aesthetic impairment, metallic taste, manufacturing challenges, and incompatibility with imaging techniques [20]. These drawbacks, together with patients’ preferences for metal-free prostheses, have encouraged researchers to investigate alternative materials [17,20].

Another option is the use of ceramic frameworks, such as zirconia, to enhance the durability of the prosthesis, preserve optimal esthetics and function [17]. However, ceramic materials have certain disadvantages, such as a lengthy manufacturing process, a high elastic modulus, and high density [21,22].

In contrast to rigid metallic and ceramic frameworks, some researchers have hypothesized that less rigid materials may distribute loads more evenly in the substructures and reduce bone resorption. Polyetheretherketone (PEEK) is a polymeric biocompatible material. The elastic modulus of PEEK is closer to that of bone; therefore, it may improve shock absorption [23]. According to previous studies, PEEK frameworks appear to provide favorable outcomes regarding the health of the supporting tissues [24,25]. 

A semi-rigid material that has gained popularity in recent years is glass fiber-reinforced composites (GFRC), which consists of a polymerized monomer matrix filled with thin glass fibers [26]. Numerous studies have suggested that GFRC demonstrates favorable performance as a framework material [27,28].

Finite element analysis (FEA) is used in dentistry for a variety of purposes, such as evaluating the stress distribution in dental prostheses, assessing material behavior, material failure, and performance in various forms of prosthetic rehabilitation. [29,30]. Static FEA is used when loads remain constant or change slowly, whereas dynamic FEA is applied when loads change rapidly or when vibrations and inertia have a significant effect on the structure’s behavior [31].

Although several previous studies have assessed the biomechanical performance of different framework and superstructure materials in full-arch ISP with bar frameworks using static FEA [7,32,33], there is limited information in the literature regarding posterior partially edentulous cases. More specifically, limited data are available concerning the stress distribution and biomechanical behavior of various framework–superstructure material combinations in posterior four-unit Toronto prostheses supported by two implants under dynamic loading conditions.

This treatment modality represents a clinically relevant option for posterior partially edentulous patients, in whom biomechanical complications may be influenced by the mechanical properties of the restorative materials used. Furthermore, the increasing application of metallic, ceramic, and polymer-based materials in contemporary implant prosthodontics highlights the importance of better understanding their biomechanical behavior.

Therefore, the present study aimed to evaluate the biomechanical behavior of posterior four-unit implant-supported prostheses supported by two implants with different framework and superstructure material combinations through dynamic finite element analysis.

## 2. Materials and Methods

### 2.1. Model Creation

A three-dimensional (3D) model of the mandible was created using Mimics software (2021 version, Materialise NV, Leuven, Belgium). Hard-tissue segmentation was used to reconstruct the 3D geometry of the jaw in order to replicate the cortical bone (2 mm thickness), cancellous bone, and a 2 mm thick mucosa [7].

Two bone-level implants (Nobel Biocare, Zurich, Switzerland) were vertically placed in the first premolar (12 mm length, 3.5 mm diameter) and second molar (10 mm length, 4.3 mm diameter) regions. Both implants were fitted with straight abutments (1.5 mm gingival height, 4.3 mm diameter). The geometries of the implants, abutments, prosthetic screws, and additional implant components were generated using manufacturer-provided data from Nobel Biocare. 

Eight ISP models were generated using four framework materials—Ti, GFRC, 3Y-TZP zirconia, and PEEK—and two superstructure materials, 5Y-TZP zirconia and resin-matrix ceramic (RMC).

Group 1. Ti framework + 5Y-TZP zirconia crown.

Group 2. Ti framework + RMC crown.

Group 3. GFRC framework + 5Y-TZP zirconia crown.

Group 4. GFRC framework + RMC crown.

Group 5. 3Y-TZP zirconia framework + 5Y-TZP zirconia crown.

Group 6. 3Y-TZP zirconia framework + RMC crown.

Group 7. PEEK framework + 5Y-TZP zirconia crown.

Group 8. PEEK framework + RMC crown.

**Figure 1:**
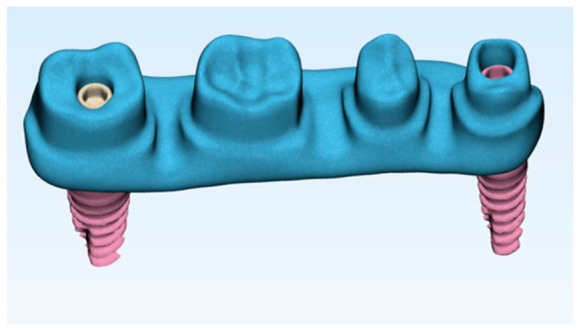
Toronto prosthesis design supported by two implants.

Frameworks and superstructures were designed using Exocad software (exocad GmbH, Darmstadt, Germany; version 3.2) in a Toronto prosthesis configuration. Connector dimensions were set at 5.0 mm width and 5.5 mm height [34,35], with rounded geometry [36]. The Toronto prosthesis was designed with a standardized mesiodistal length of 36 mm to ensure anatomical consistency [37] (Figure 1).

All models, except those with Ti frameworks, were fitted with multi-unit Ti bases (4.5 mm height, 2.42 mm width) positioned above the abutments and connected to the frameworks using 1.25 mm diameter prosthetic screws. To provide space for cementation between the multi-unit Ti base and the framework, a 25 μm cement space was defined. The superstructures were designed as monolithic single crowns with a uniform thickness of 1.5 mm [38,39]. A 25 μm cement space was defined between the framework and the crowns [40]. However, the cement layers were not explicitly modeled as separate finite element components and all corresponding interfaces were assumed to be perfectly bonded. All materials were assumed to be homogeneous, isotropic, linearly elastic. The mechanical properties of the materials used in the present study are summarized in Table 1.

All files were exported in Standard Tessellation Language (STL) format from Mimics and Exocad software and then converted to Standard for the Exchange of Product data (STEP) format using Geomagic software (2022 version, 3D Systems, Rock Hill, SC, USA). The converted data were then imported into Abaqus software (2024 version, Dassault Systèmes, Vélizy-Villacoublay, France), where the models’ nodes and meshes were generated, and loading conditions were applied.

### 2.2. Meshing

A high-density tetrahedral 3D mesh was used for critical components and regions (peri-implant area, implant–bone interface, frameworks, and implant screws), whereas a coarser mesh (larger elements) was applied in less critical regions. 

A mesh convergence test was conducted using Abaqus software (Dassault Systèmes, Vélizy-Villacoublay, France) to verify the accuracy and reliability of the finite element models employed in this study. The purpose of this test was to identify an optimal number of mesh elements capable of producing reliable numerical results while maintaining an error rate below 5% [17].

Several finite element models containing different numbers of mesh elements were generated and evaluated under identical loading and boundary conditions to ensure consistency in the analysis. Von Mises stress values were selected as the primary evaluation criterion because of their widespread use in assessing the biomechanical behavior of ISP systems.

The von Mises stress values obtained from each model were compared to evaluate the effect of mesh refinement. As the number of mesh elements increased, the changes in von Mises stress values gradually decreased and the results became more stable, indicating mesh convergence. The calculated stress values ranged from approximately 1.4 MPa in the model with 100,000 elements to 1.9 MPa in the model with 715,000 elements.

**Figure 2:**
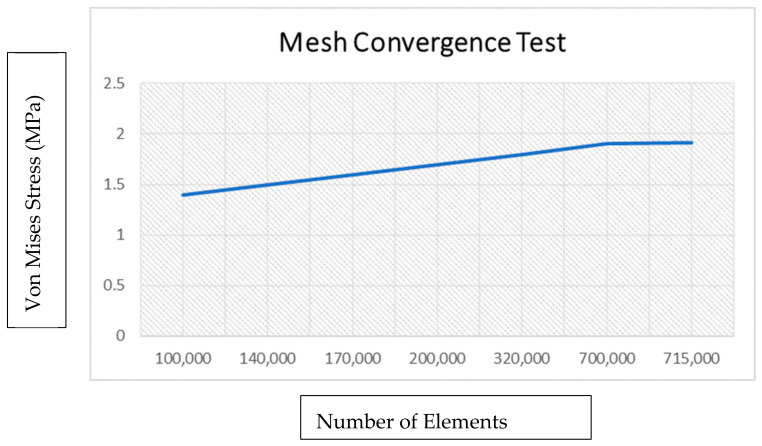
Results of the mesh convergence test.

Only minor changes were detected after approximately 715,000 elements, indicating that the selected mesh density was likely adequate for the purposes of the present study, and further increases in the number of mesh elements had little effect on the obtained results (Table 2 and Figure 2). Therefore, the selected mesh configuration was regarded as reliable and suitable for the final FEA.

**Table 2:** Number of mesh elements and corresponding von Mises stress values obtained from the mesh convergence test.

Number of Elements	Von Mises Stress (MPa)
100,000	1.4
140,000	1.5
170,000	1.6
200,000	1.7
320,000	1.8
700,000	1.9
715,456	1.9

The final finite element model consisted of 715,456 tetrahedral elements and 1,362,760 nodes. These parameters are important for evaluating the biomechanical behavior of the system. The total numbers of elements and nodes in the final model are presented in Table 3.

### 2.3. Loading

**Figure 3:**
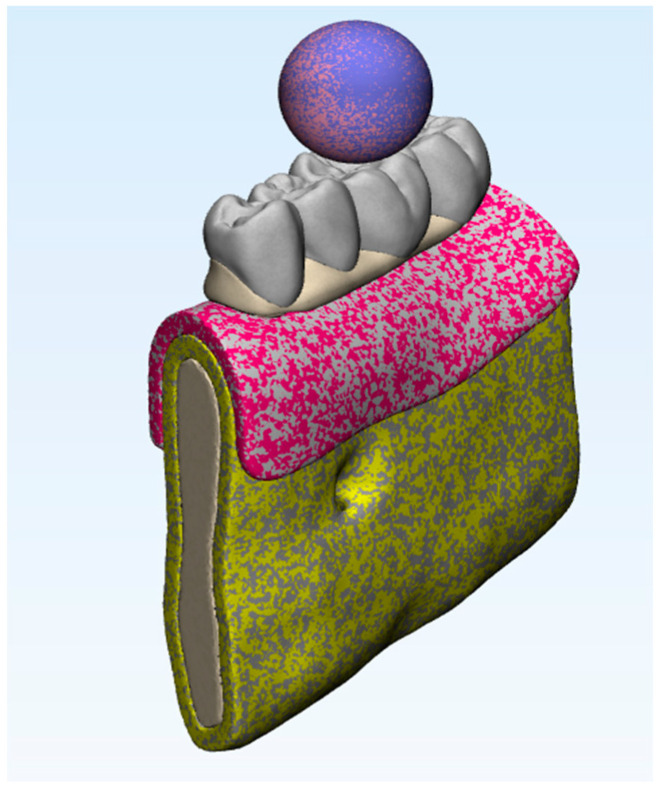
Spherical solid body simulating the food bolus positioned on the left first molar region.

In the present study, a transient dynamic FEA with quasi-static loading conditions was performed. The inferior border of the mandibular model was fixed in all directions (X, Y, and Z axes) to simulate mandibular stabilization during loading and prevent rigid body motion. A dynamic occlusal load of 150 N was gradually applied to the first molar over a chewing cycle of 0.3 s using a spherical solid body (12 mm in diameter) to simulate the food bolus [7] (Figure 3). The spherical body was intended to provide a standardized loading condition rather than to reproduce the exact mechanical behavior of a specific food material. The selected material properties were based on previously published finite element study [7].

Complete osseointegration between the implant and bone surfaces was assumed. In addition, all contact interfaces between the components were modeled as perfectly bonded, assuming complete seating and adaptation without frictional contact or relative movement along the interfaces, thereby simulating mechanical stability of the prosthetic assembly under loading.

## 3. Results

The reported von Mises stress and displacement values represent the maximum values obtained during the 0.3 s loading interval.

### 3.1. Stress Distribution

**Figure 4:**
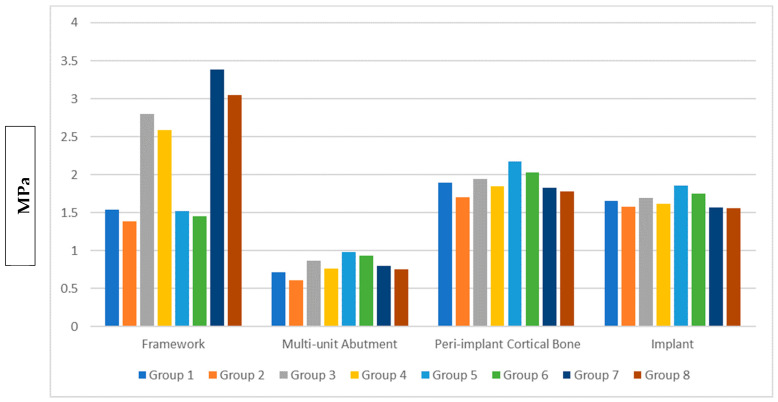
Graphical comparison of von Mises stress values (MPa) in the frameworks, multi-unit abutments, peri-implant cortical bone, and implants across different groups.

The von Mises stress criterion was used to evaluate stress distribution. The von Mises stress values in the frameworks, abutments, peri-implant cortical bone, and implants for each group are shown in Table 4 and graphically illustrated in Figure 4.

#### 3.1.1. Stress Distribution Across Frameworks

**Figure 5:**
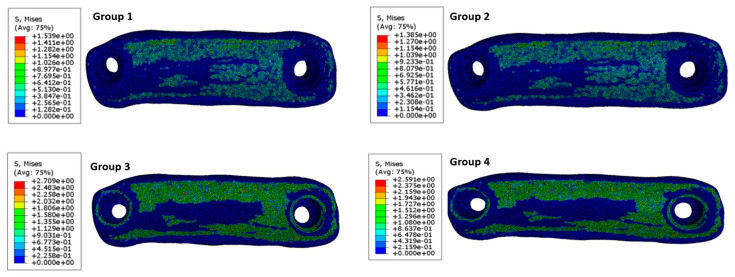
Stress distribution patterns within the frameworks in Groups 1, 2, 3 and 4.

**Figure 6:**
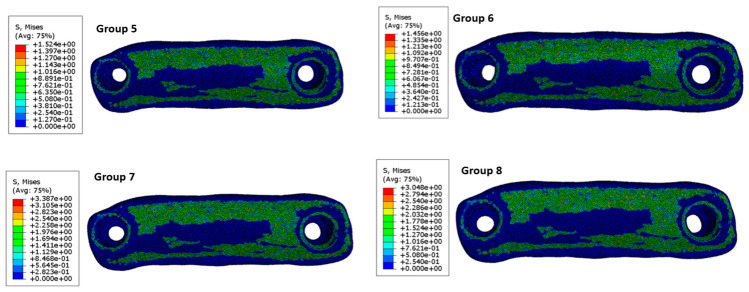
Stress distribution patterns within the frameworks in Groups 5, 6, 7 and 8.

Among the evaluated frameworks, the highest stress value was observed in Group 7, whereas the lowest value was recorded in Group 2. RMC crowns consistently demonstrated lower von Mises stress values than 5Y-TZP zirconia crowns across all groups. In all groups, the peak stress concentration was observed in the first molar region (Figure 5 and Figure 6). 

#### 3.1.2. Stress Distribution in Abutments

The highest and lowest von Mises stress values in the abutments were observed in Group 5 and Group 2, respectively. Among the framework materials, zirconia frameworks exhibited the highest stress values in the abutments, followed by GFRC, PEEK, and Ti.

**Figure 7:**
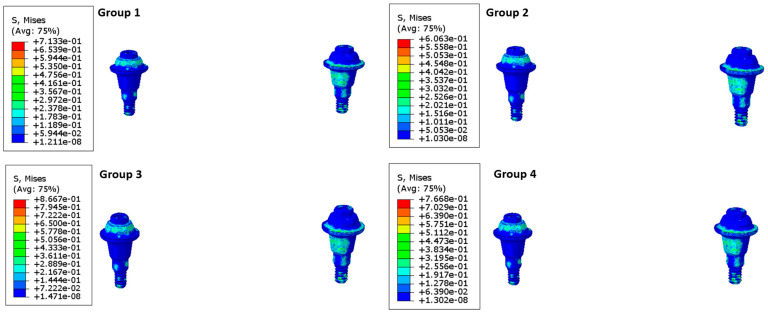
Stress distribution patterns within the abutments for Groups 1, 2, 3 and 4.

**Figure 8:**
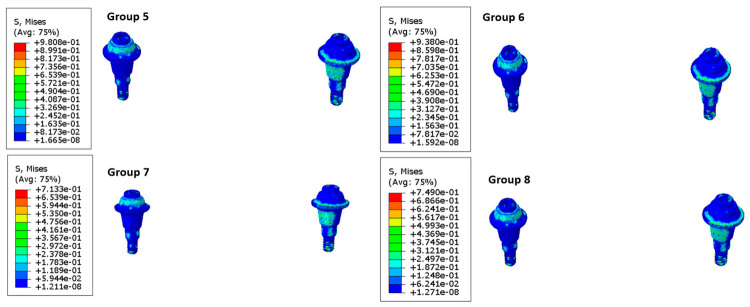
Stress distribution patterns within the abutments for Groups 5, 6, 7 and 8.

Furthermore, within each group, RMC crowns consistently demonstrated lower stress values than 5Y-TZP zirconia crowns. Among all groups, the highest stress concentration was observed at the cervical (neck) region of the anterior multi-unit abutment, whereas peak stress in the posterior abutment was localized in the midsection of the abutment body (Figure 7 and Figure 8).

#### 3.1.3. Stress Distribution in Peri-Implant Cortical Bone

**Figure 9:**
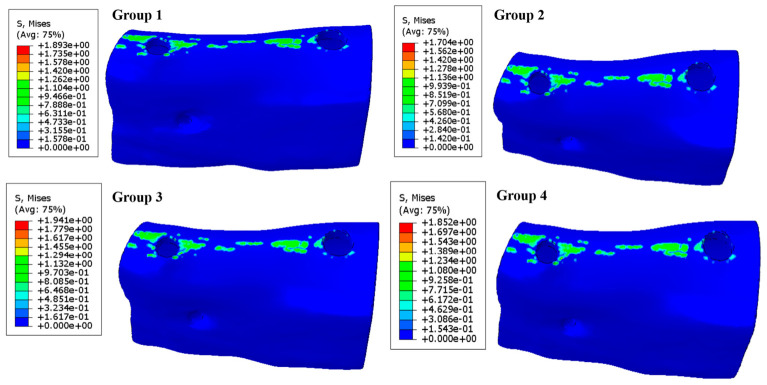
Stress distribution patterns within the peri-implant cortical bone for Groups 1, 2, 3 and 4.

**Figure 10:**
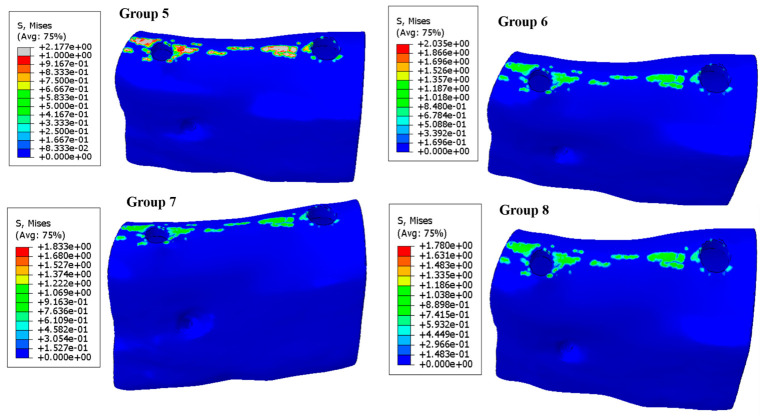
Stress distribution patterns in the peri-implant cortical bone for Groups 5, 6, 7 and 8.

Among all groups, the highest von Mises stress value in the peri-implant cortical bone was observed in Group 5, whereas the lowest was recorded in Group 2. Peak stress concentrations were predominantly located in the first molar region and around the abutments across all groups. Similar to the other components analyzed, RMC crowns consistently demonstrated lower stress values than 5Y-TZP zirconia crowns (Figure 9 and Figure 10).

#### 3.1.4. Stress Distribution in Implants

**Figure 11:**
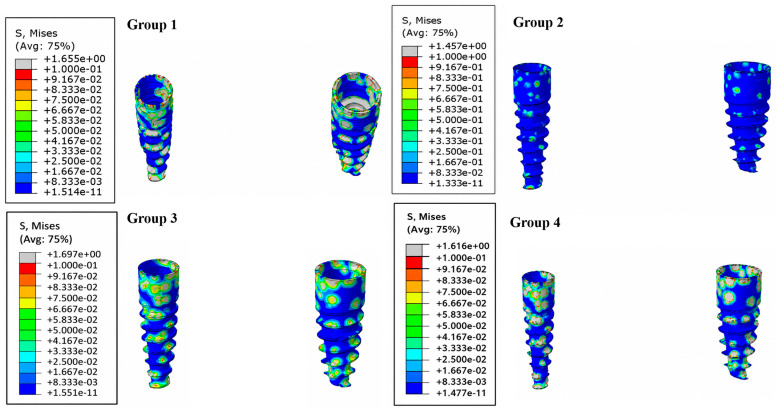
Stress distribution patterns within the implants in Groups 1, 2, 3 and 4.

**Figure 12:**
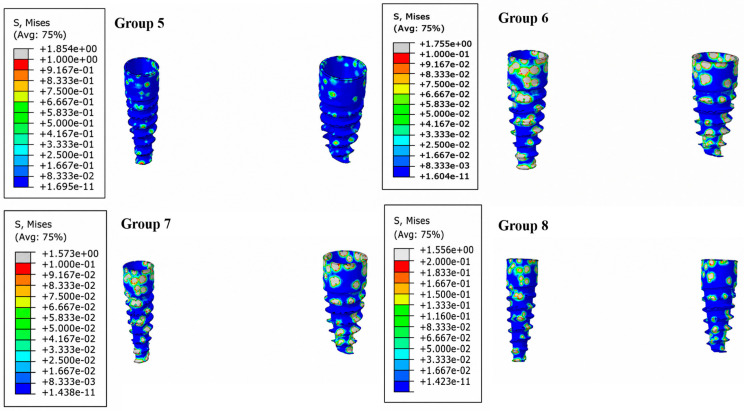
Stress distribution patterns within the implants in Groups 5, 6, 7 and 8.

The highest von Mises stress value was observed in Group 5, whereas the lowest was recorded in Group 2. Peak stress concentrations within the implant body were predominantly concentrated in the cervical (neck) region across all groups. In agreement with the other components evaluated, RMC crowns showed consistently lower stress levels than 5Y-TZP zirconia crowns across all groups (Figure 11 and Figure 12).

### 3.2. Displacement

**Figure 13:**
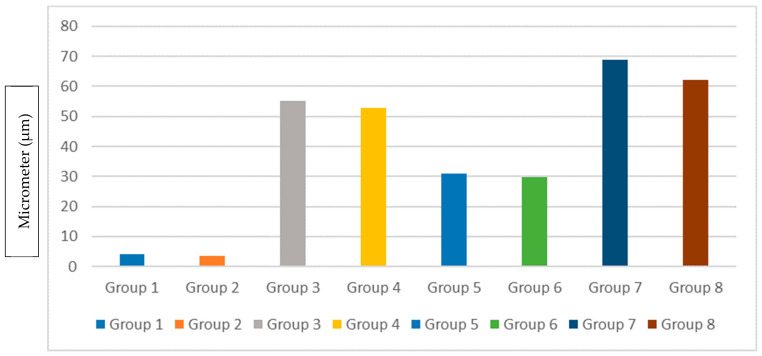
Graphical comparison of displacement in the frameworks.

Framework displacement values across the different groups are shown in Table 5 and Figure 13. Among the framework materials, PEEK demonstrated the highest displacement values, followed by GFRC, 3Y-TZP zirconia, and Ti.

## 4. Discussion

In this study, the biomechanical behavior of various framework and superstructure material combinations in a four-unit implant-supported Toronto prosthesis supported by two implants was evaluated. The present study used dynamic FEA, a method that may more accurately replicate the time-varying nature of masticatory loading, an aspect that has not been extensively investigated in many static FEA studies [17].

The Toronto prosthesis combines the advantages of both cement- and screw-retained restorations, allowing easy retrievability of both the abutment and prosthesis, facilitating the removal of excess cement, and improved prosthetic longevity with reduced maintenance costs, without increasing the risk of porcelain fracture or screw loosening [14,15,16]. The rehabilitation of partially or fully edentulous patients is often complicated by poor bone quality, insufficient bone volume due to prolonged edentulism, and anatomical limitations, particularly in the posterior region [41,42]. The Toronto prosthesis may represent a less invasive alternative to extensive augmentation procedures, while allowing the use of esthetic yet brittle restorative materials [41,42,43].

Considering these clinical challenges, limited space for implant placement, poor bone quality, and the presence of bone defects may restrict the use of an optimal number of implants [8,44]. Although alternative approaches, such as short implants or bone augmentation procedures, are available, they are often associated with increased cost, extended treatment time, and higher postoperative morbidity [44,45]. In such cases, implant-supported bridge restorations represent a viable treatment option. Previous studies [36,46,47] have reported favorable outcomes for four-unit ISP supported by 2 implants, demonstrating their clinical effectiveness and clinical applicability.

From a biomechanical perspective, the design of the prosthetic framework plays an important role in stress distribution. A round connector cross-sectional geometry was selected based on previous studies demonstrating its superior fatigue resistance compared with trapezoidal or square designs [36,48,49]. 

In the present study, von Mises stress was selected as the primary biomechanical parameter because it has been widely applied in implant-related FEA, thereby enabling standardized comparison with previously published studies in the literature. This criterion has frequently been applied to evaluate stress distribution in prosthetic components, implants, screws, supporting structures, and peri-implant bone tissues [35,50,51]. Furthermore, von Mises analysis provides a single equivalent stress value under complex multiaxial loading conditions, facilitating the overall biomechanical comparison among the investigated models and structures. Since the primary objective of the present study was to compare general stress distribution patterns rather than predict material fracture or biological failure, von Mises stress was considered an appropriate and practical parameter for analysis. However, since cortical bone and ceramic materials exhibit complex mechanical behavior and may not behave as ideal ductile materials, the interpretation of von Mises stress values should be approached with caution. Therefore, the results of the present study should be interpreted within the assumptions and inherent limitations of FEA and material simplification [52].

Several previous FEA studies have investigated the influence of different framework materials on stress distribution and biomechanical behavior in implant-supported prostheses [7,35,50,51,53]. Shash et al. [35] compared five framework materials in an All-on four prosthesis—alumina, zirconia, Ti, fiberglass-reinforced resin (FRR), and PEEK—and reported that zirconia frameworks generated higher stress concentrations in implants, abutments, and surrounding bone structures, whereas PEEK frameworks demonstrated comparatively lower stress values. Similarly, Haroun and Ozan [7] reported lower stress values in cortical bone and implants when PEEK frameworks were used in comparison with more rigid framework materials. Comparable findings were also reported by Tahilramani et al. [50], who observed lower implant and cortical bone stress values with PEEK frameworks than with zirconia frameworks in a 3-unit ISP. This behavior may be attributed to differences in the elastic modulus of the evaluated framework materials. Materials with a higher Young’s modulus may show less deformation and tend to transfer greater stress to the abutment, implant, and surrounding bone structures. In contrast, materials with a lower elastic modulus may allow for greater stress absorption and more distributed stress patterns, which may contribute to reducing the magnitude of stress transmitted to the implant components and adjacent bone tissues. Similar biomechanical behaviors have been reported in several FEA studies investigating the influence of different framework materials in ISP [7,35,50].

In contrast to the findings of the present study, Sirandoni et al. [53] reported lower stress values within the framework when PEEK was used, while higher stress values were observed in the surrounding bone structures. This difference may be related to variations in the prosthetic design and material configuration used in the analyses. In the study by Sirandoni et al. [53], only the framework material was evaluated without the addition of a superstructure material. In the present study, the combined use of framework and superstructure materials may have influenced stress distribution pattern and contributed to lower stress transmission to the surrounding bone structures when PEEK frameworks were used.

RMC crowns consistently demonstrated lower stress values than 5Y-TZP zirconia crowns. RMCs are primarily composed of a polymeric matrix reinforced with inorganic refractory constituents, including porcelain, glass, ceramic, and glass-ceramic [54]. Compared with 5Y-TZP zirconia, RMCs exhibit greater fatigue resistance, enhanced reliability, improved internal adaptation, and a lower modulus of elasticity [55].

In addition to stress analysis, displacement is an important parameter to evaluate the mechanical behavior of prosthetic frameworks. Displacement represents the positional change in an object relative to a defined reference point; in FEA, this reference is commonly considered the origin of the model’s coordinate system [17]. In the current study, the PEEK framework exhibited the highest displacement values, followed by the GFRC frameworks. These findings are consistent with previous studies suggesting that polymer-based materials may undergo greater deformation and displacement than metallic and ceramic alternatives [17,56,57].

Although screw preload was not evaluated in the present FEA study, the relatively greater displacement and deformation observed in low-elastic-modulus materials such as PEEK and GFRC may indicate altered load transfer patterns to the prosthetic and abutment screw components [58,59]. The shock-absorbing characteristics of these materials could influence the biomechanical response of the implant-supported system. However, since screw preload, frictional contact, and thread engagement were not simulated, any possible association with screw loosening should be considered hypothetical and interpreted with caution.

Among the factors affecting the biomechanical behavior of ISP, occlusion has been identified as a critical factor [9,36]. The selection of an appropriate occlusal scheme, along with the proper adjustment of occlusal contacts, plays an important role in determining the magnitude and distribution of forces transmitted to the prosthetic components [7,9]. The first molar region was selected as the loading area because it represents a major load-bearing area during mastication and is exposed to the greatest occlusal forces within the posterior mandibular region [60,61]. In the present study, a spherical solid body contacting the cusps of the mandibular first molar was employed to minimize the complexity of occlusal adjustment across different models and to ensure methodological standardization. Additionally, this approach enabled the application of forces in vertical, horizontal, and oblique directions [7].

Although the present study aimed to simulate clinical conditions as closely as possible, the findings should be interpreted within the limitations of FEA. The current model did not include experimental validation, patient-specific anatomical variability, fatigue simulation, thermal and aging effects, or biological remodeling responses. In addition, the geometries of the cortical bone and mucosa were simplified, and uniform thicknesses were assumed throughout the model. Complete osseointegration of the implants was also assumed, which may not fully represent clinical conditions.

Furthermore, non-titanium framework groups were modeled with multi-unit Ti bases, whereas the titanium framework groups were directly connected to the implant components. Although this configuration was selected to reflect commonly applied clinical restorative protocols, it may have introduced additional differences related to the connection configuration between the evaluated groups.

All materials evaluated in this study were considered homogeneous, isotropic, and linearly elastic. However, GFRC, PEEK, and RMC may demonstrate anisotropic and viscoelastic behavior under clinical conditions. These assumptions were adopted to simplify the computational analysis, reduce modeling complexity, and maintain methodological consistency with previous dental FEA studies. Moreover, the use of standardized material assumptions enabled direct comparison among the investigated materials and facilitated comparison with previous FEA studies.

It is important to note that all interfaces were modeled as perfectly bonded, assuming complete seating and mechanical stability of the prosthetic assembly under loading. This simplification was implemented to ensure numerical stability and to focus on the comparative stress distribution within the prosthetic components. However, the current model does not simulate frictional contact, deformation of the cement layer, screw-joint behavior, or potential interfacial micromotion. These factors may affect the biomechanical response and should be addressed in future investigations for a more clinically realistic simulation.

Future clinical and experimental studies incorporating more clinically realistic simulation conditions are required to provide a better understanding of the biomechanical behavior and long-term mechanical performance of four-unit implant-supported Toronto prostheses supported by two implants.

## 5. Conclusions

Within the limitations of the present study, the following conclusions were drawn:

1. Among all evaluated material combinations, the Ti framework combined with RMC crowns demonstrated the lowest stress values in the framework, multi-unit abutment, implant, and peri-implant cortical bone, while also exhibiting the lowest framework displacement.
2. Across all tested framework materials, RMC crowns demonstrated lower stress values than 5Y-TZP zirconia crowns.
3. Framework materials with a higher Young’s modulus tended to demonstrate lower deformation while transferring greater stress to implant components and peri-implant bone structures. In contrast, materials with a lower elastic modulus tended to show greater deformation and more distributed stress patterns under the modeled conditions, although greater displacement and deformation were also observed.

## Figures and Tables

**Table 1:** Mechanical properties of the materials.

Material	Elastic Modulus (GPa)	Poisson’s Ratio
Ti	110	0.35
GFRC	26	0.2
3Y-TZP Zirconia	214	0.30
PEEK	4	0.33
5Y-TZP Zirconia	205	0.30
RMC	29.8	0.30
Cortical Bone	13.7	0.35
Cancellous Bone	1.4	0.30
Mucosa	1	0.37
Food Bolus	84.1	0.33

**Table 3:** The total number of elements and nodes of the model.

Model	Number of Elements	Number of Nodes
Superstructures	80,620	153,176
Toronto Prostheses	84,318	160,203
Multi-unit Abutments	64,086	121,762
Prosthetic Screws	30,767	58,456
Mucosa	51,792	98,403
Cortical Bone	102,535	194,815
Spongy Bone	96,934	184,175
Implants	93,058	176,807
Multi-unit Ti base	34,351	65,268
Food Bolus	76,784	145,889

**Table 4:** Von Mises stress values (MPa) in the frameworks, multi-unit abutments, peri-implant cortical bone, and implants among different groups.

Group	Framework Stress	Abutment Stress	Peri-Implant Cortical Bone Stress	Implant Stress
Group 1	1.539	0.713	1.893	1.655
Group 2	1.385	0.606	1.704	1.457
Group 3	2.709	0.867	1.941	1.697
Group 4	2.591	0.767	1.852	1.616
Group 5	1.524	0.981	2.177	1.854
Group 6	1.456	0.938	2.035	1.755
Group 7	3.387	0.802	1.833	1.573
Group 8	3.048	0.749	1.780	1.556

**Table 5:** Displacement values (µm) of the framework among different groups.

Group	Framework Displacement (µm)
Group 1	4.09
Group 2	3.68
Group 3	55.19
Group 4	52.77
Group 5	31.04
Group 6	29.66
Group 7	68.98
Group 8	62.08

## Data Availability

The data presented in this study are available on request from the corresponding author upon reasonable request due to privacy restrictions.

## Abbreviations

The following abbreviations are used in this manuscript:

ISP	Implant-Supported Prostheses
FEA	Finite Element Analysis
3D	Three-Dimensional
Ti	Titanium
GFRC	Glass Fiber Reinforced Composite
PEEK	Polyether Ether Ketone
RMC	Resin Matrix Ceramic
STL	Standard Tessellation Language
STEP	Standard for the Exchange of Product Data
FRR	Fiberglass-reinforced Resin
µm	Micrometer
